# Psychostimulant-Induced Testicular Toxicity in Mice: Evidence of Cocaine and Caffeine Effects on the Local Dopaminergic System

**DOI:** 10.1371/journal.pone.0142713

**Published:** 2015-11-11

**Authors:** Candela R. González, Betina González, María E. Matzkin, Javier A. Muñiz, Jean Lud Cadet, Edgar Garcia-Rill, Francisco J. Urbano, Alfredo D. Vitullo, Veronica Bisagno

**Affiliations:** 1 Centro de Estudios Biomédicos, Biotecnológicos, Ambientales y Diagnóstico (CEBBAD), Universidad Maimónides, Ciudad Autónoma de Buenos Aires, Buenos Aires, Argentina; 2 Instituto de Investigaciones Farmacológicas (Universidad de Buenos Aires–Consejo Nacional de Investigaciones Científicas y Técnicas), Ciudad Autónoma de Buenos Aires, Buenos Aires, Argentina; 3 Instituto de Biología y Medicina Experimental (Universidad de Buenos Aires–Consejo Nacional de Investigaciones Científicas y Técnicas), Ciudad Autónoma de Buenos Aires, Buenos Aires, Argentina; 4 NIDA Intramural Program, Molecular Neuropsychiatry Research Branch. Baltimore, Maryland, United States of America; 5 Center for Translational Neuroscience, Department of Neurobiology and Developmental Sciences, University of Arkansas for Medical Sciences, Little Rock, Arkansas, United States of America; 6 Laboratorio de Fisiología y Biología Molecular, Instituto de Fisiología, Biología Molecular y Neurociencias (Universidad de Buenos Aires–Consejo Nacional de Investigaciones Científicas y Técnicas), Ciudad Autónoma de Buenos Aires, Buenos Aires, Argentina; University of Hyderabad, INDIA

## Abstract

Several organ systems can be affected by psychostimulant toxicity. However, there is not sufficient evidence about the impact of psychostimulant intake on testicular physiology and catecholaminergic systems. The aim of the present study was to further explore potential toxic consequences of chronic exposure to cocaine, caffeine, and their combination on testicular physiology. Mice were injected with a 13-day chronic binge regimen of caffeine (3x5mg/kg), cocaine (3×10mg/kg), or combined administration. Mice treated with cocaine alone or combined with caffeine showed reduced volume of the seminiferous tubule associated to a reduction in the number of spermatogonia. Cocaine-only and combined treatments induced increased lipid peroxidation evaluated by TBARS assay and decreased glutathione peroxidase mRNA expression. Importantly, caffeine-cocaine combination potentiated the cocaine-induced germ cell loss, and induced pro-apoptotic BAX protein expression and diminished adenosine receptor A1 mRNA levels. We analyzed markers of dopaminergic function in the testis and detected the presence of tyrosine hydroxylase (TH) in the cytoplasm of androgen-producing Leydig cells, but also in meiotic germs cells within seminiferous tubules. Moreover, using transgenic BAC-Drd1a-tdTomato and D2R-eGFP mice, we report for the first time the presence of dopamine receptors (DRs) D1 and D2 in testicular mouse Leydig cells. Interestingly, the presence of DRD1 was also detected in the spermatogonia nearest the basal lamina of the seminiferous tubules, which did not show TH staining. We observed that psychostimulants induced downregulation of DRs mRNA expression and upregulation of TH protein expression in the testis. These findings suggest a potential role of the local dopaminergic system in psychostimulant-induced testicular pathology.

## Introduction

Addictions to licit and illicit drugs represent chronic relapsing brain disorders that affect circuits involved in reward, motivation, memory, and decision-making. Drug-induced pathological changes in these brain regions are associated with characteristic behaviors that endure despite adverse biopsychosocial consequences [1]. Drugs of abuse also can lead to dysfunction of multiple organ systems [2]. Particularly, cocaine can negatively impact testicular physiology with direct consequences on the spermatogenic process [3]. Cocaine exerts adverse effects in the testis, producing morphological changes, testicular antioxidant depletion, and apoptosis of male germ cells that lead to reduced sperm production both in humans and rodents [4–7]. Although testicular binding sites for cocaine have been demonstrated [8], the mechanism by which cocaine and other psychostimulants affect testicular physiology has not been fully elucidated. Notably, recent reports described cocaine-mediated epigenetic changes (i.e. increased histone acethylation) leading to reprogramming of the male germline and to paternal transmission of cognitive and addiction-related phenotypes to the progeny [9,10].

Extensive evidence suggests that the reinforcing effects of cocaine on the central nervous system (CNS) involve inhibition of monoamine transporters, which restrain monoamine lifetime after release [11,12]. Cocaine is an inhibitor of the dopamine (DA), serotonin (5-HT), and norepinephrine (NE) transporters [11]. Through this mechanism of action cocaine can induce increases in monoamine volume transmission by affecting reuptake [11]. Also, cocaine-mediated DA transporter (DAT) blockade causes elevated extracellular DA levels and dopamine receptor (DR) downregulation, linked to addictive behavior [13,14]. Cocaine also binds to several other proteins, including neurotransmitter receptors, plasma proteins, voltage gated ion channels, and metabolic enzymes [15]. It has been extensively documented that an increase in DA levels is linked to an increase in reactive oxygen species (ROS) production and neuronal degeneration [16]. The involvement of oxidative stress in cocaine-induced toxicity has also been reported in peripheral organs and systems, including heart, liver, kidney, and testis [6,17]. In both central and peripheral systems, depletion of cellular antioxidant defenses and impairment of mitochondrial respiration have been considered important causes of ROS production and subsequent cell death mediated by cocaine, confirming the link between cocaine toxicity and redox-mediated pathways [17,18].

Several studies indicate that concomitant consumption of caffeine with other psychostimulant drugs such as cocaine can profoundly alter drug response and induce adverse effects [19,20]. The interaction between caffeine and cocaine has clinical relevance since caffeine is one of the most common adulterants of cocaine [21,22]. Caffeine, at low doses, acts as an antagonist of adenosine A1 and A2a receptors, which negatively modulate the signaling of DRD1 and DRD2, respectively. Therefore, the stimulating effects of caffeine are due to indirect reinforcement of dopaminergic transmission [23].

The effects of cocaine on peripheral systems have not been thoroughly explored despite growing evidence for peripheral sources and actions of DA, and the widespread expression of DRs in peripheral tissues [24]. In the testes, DA appears to be located in the wall of seminiferous tubules and interstitial cells [25]. Mayerhoffer et al. [26,27] studied testicular catecholaminergic innervation and found a dual input: one provided by extrinsic sympathetic innervation and another involving neuron-like cells located in the interstitial compartment. Different testicular actions of catecholamines modulating testosterone production have been described [28,29]. Moreover, tyrosine hydroxylase (TH), the rate-limiting enzyme of catecholamine synthesis, was found in Leydig cells, neuron-like cells and nerve fibers [27, 30, 31]. In addition, DAT was found in neuron-like cells and nerve fibers in human testicular tissue [27]. To date, only DRD2 has been described in testicular germ cells, suggesting paracrine actions of DA in the spermatogenic process [32]. On the other hand, there is limited information available on the adenosinergic system in the testis, despite the fact that adenosine receptors have been found in both somatic and germ cells [33].

Although the pharmacological targets of psychostimulant drugs on dopaminergic/adenosinergic pathways in the CNS have been extensively documented, there is insufficient evidence of their impact on peripheral dopaminergic systems. It is important to note that the testis is the second organ after the brain presenting the highest cocaine concentrations after radioactive cocaine administration [34]. Therefore, the aim of the present study was to further explore physiological and potential toxic consequences of chronic exposure to cocaine, caffeine, and their combination on testicular physiology. Here, we report that caffeine can potentiate cocaine-induced germ cell loss associated with increased expression of pro-apoptotic BAX, and reduced LHR protein and A1 mRNA expression. We also provide evidence that caffeine and cocaine treatment is able to downregulate DR mRNA, and increase TH protein expression. To our knowledge, this is the first study showing psychostimulant effects on dopaminergic markers expression in the testis.

## Materials and Methods

### Animals

Male C57BL/6 mice (10–12 weeks old) from the School of Exact and Natural Sciences of the University of Buenos Aires (UBA) were housed in a light- and temperature-controlled room. Mice had free access to food and water. Principles of animal care were followed in accordance with “Guidelines for the Care and Use of Mammals in Neuroscience and Behavioral Research” (National Research Council, 2003) and approved by IACUC Committee of the Faculty of Pharmacy and Biochemistry, Universidad de Buenos Aires (Protocol Number: EXP_UBA N° 40944/20151). Mice were euthanized by cervical dislocation performed by an individual proficient in this technique.

To detect the expression of DRs in testicular tissue, male C57BL/6J inbred bacterial artificial chromosome (BAC)-transgenic mice Drd1a-tdTomato and D2R-enhanced green fluorescent protein (eGFP) were used (10–12 weeks old, N = 3 of each genetic background). These transgenic mice carry a BAC transgene with the promoter and regulatory sequences of DRD1 and DRD2 controlling the expression of fluorescent reporters [35].

### Pharmacological and Physiological Procedures

**Fig 1:**
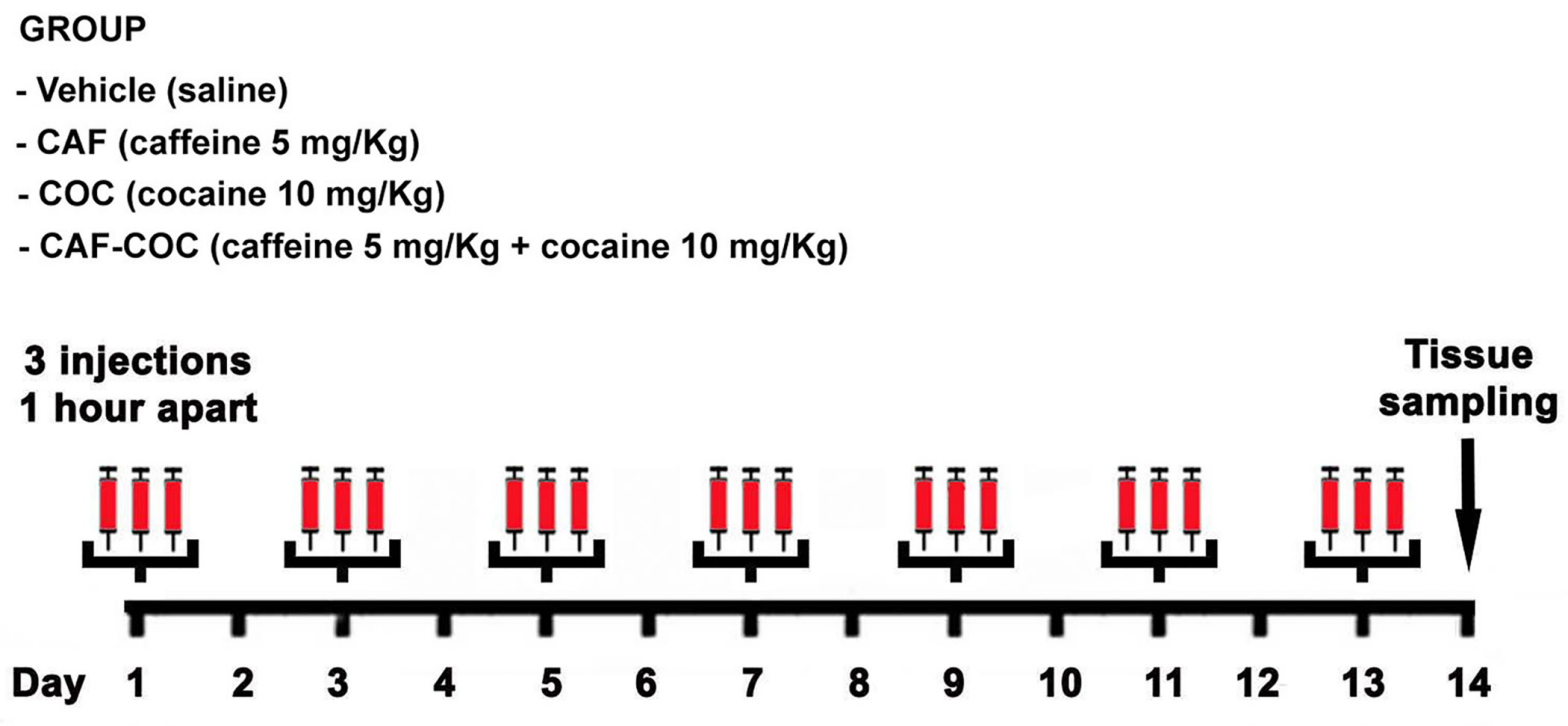
Schematic representation of the experimental treatments. Male C57BL/6 mice were subjected to an intermittent chronic treatment (one day on/one day off for 13 days) with caffeine 5 mg/Kg (CAF), cocaine 10 mg/kg (COC) and their combination (5 mg/kg caffeine + 10 mg/kg cocaine, CAF-COC) in a binge protocol: 3 i.p. injections per day, 1 hour apart. Tissue samples were taken 24 hr after the last binge.

Cocaine hydrochloride and caffeine (Sigma-Aldrich, St Louis, MO) were administered in a *binge-like* regimen of three i.p. injections per day, 1 hr apart. Animals were assigned to four different groups: COC (Cocaine-only 3 x 10 mg/kg), CAF (Caffeine-only 3 x 5 mg/kg), CAF-COC (Combined 3 x cocaine 10 mg/kg + Caffeine 5 mg/kg), both dissolved in sterile saline and co-administered in a single injection) or Vehicle (3 x sterile saline), see Fig 1. The binge pattern of cocaine administration in rodents is a model of human consumption, which mimics the behavioral and neurochemical changes documented in patient populations. Binge cocaine administration (defined as the ingestion of significant amounts of drug over a short period of time), it is considered a typical use pattern in cocaine abusers and can result in successive “spikes” of dopamine concentration, as shown in animal models [36]. We and others have shown detrimental effects of cocaine administrated in a *binge*-like protocol (3 x 15 mg/kg day) on brain areas involved in reward [37], motor, and sensory functions [38,39]. Since the aim of the present study was to explore potential additive/synergistic effects of a combination of psychostimulants (i.e. caffeine + cocaine), we selected a lower dose of cocaine (10 mg/kg) that potentiates locomotor activity without eliciting behavioral sensitization [40]. Moreover, this dose, when combined with caffeine, is known to elicit locomotor sensitization [40]. The testes were removed and immediately stored at -70°C for molecular analyses, or fixed in 4% paraformaldehyde (PFA) and embedded in paraffin blocks for immunohistochemistry. For detection of DR expression in BAC-Drd1a-tdTomato and D2R-eGFP mice, testes were removed and fixed in PFA 4% for 24 hr, cryopreserved in 30% sucrose (for 24 hr), and sliced with a cryostat (12 μm) for immunofluorescence detection.

### Testis morphometry

Testis morphometry was evaluated as previously described in Gonzalez et al. [41]. Fixed testes were embedded in paraffin and serially cut in 5-μm-thick sections, mounted onto cleaned coated-slides, dewaxed in xylene, rehydrated in graded alcohols, washed in water, and processed for routine haematoxylin–eosin staining. For each specimen, at least three to five slides were stained for general histology inspection. The volume densities of the testicular tissue components were determined by light microscopy using a 441-intersection grid placed in the X400 ocular of a light microscope. Briefly, 15 fields chosen randomly (6615 points) were scored for each animal at X400 magnification. Technical artifacts were rarely seen and were not considered in the total number of points used to obtain volume densities. Points were classified as one of the following: seminiferous cords, comprising tunica propia, epithelium, and lumen; Leydig cells; blood vessels and lymphatic spaces and connective tissue. The volume of each component of the testis was determined as the product of the volume density and testis volume. The results of the testicular proportions were expressed as percentages.

### Immunohistochemistry

Mounted paraffin sections (5 μm) were dewaxed in xylene, rehydrated in graded alcohols and washed in water. Sections were subjected to antigen retrieval (20 min at 96°C in 10 nmol/L citrate buffer, pH 6.0). Endogenous peroxidase activity was inhibited using 0.5% v/v H_2_O_2_/methanol for 20 min at room temperature. Tissue sections were blocked for 30 min with 1.5% normal goat serum in phosphate-buffered saline (PBS), and incubated overnight at 4°C with primary antibody (1:100 diluted rabbit anti-DAZ-L, Ab34139, Abcam, UK; 1:50 diluted rabbit anti-TH, Pel Freez, USA). After three rinses in PBS, sections were incubated for 1 hr at room temperature with appropriate 1:200 diluted biotinylated secondary antibodies (Vector Labs, UK). After further washing in PBS, sections were incubated for 30 min with 1:100 diluted streptavidin–peroxidase complexes (ABC kit, Vector Labs, UK). Sections were washed twice with PBS and development of peroxidase activity was achieved with 0.05% w/v 3,3-diaminobenzidine and 0.1% v/v H_2_O_2_ in Tris-HCl. Sections were finally washed with distilled water. Negative controls were processed simultaneously by omitting the primary antibody or pre-absorbing the primary antibody with specific synthetic peptides. Positively stained cells for DAZL were counted in single sections using an Olympus BX40 microscope at X1000 magnification. Approximately, 400 spermatogonia were counted per slide. Spermatogonia were identified within the cords, according to their large round nuclei (larger than those of Sertoli cells) and distinct cytoplasm.

### Thiobarbituric acid-reactive substances (TBARS) assay

Phospholipid oxidation was determined by the colorimetric assay of TBARS. Testes were resuspended with PBS containing 0.4% w/v butylated hydroxytoluene on ice and then disrupted by ultrasonic irradiation. An aliquot (25 μl) of total testis extract was added to 175 μl mixed reaction solution (0.15% w/v SDS, 0.5 N HCl, 0.75% w/v phosphotungstic acid and 0.175% w/v 2-thiobarbituric acid). The mixture was heated in a boiling water bath for 45 min. TBARS were extracted with 200 μl of n-butanol. After a centrifugation at 10000×g for 5 min at 4°C, the absorbance at 532 nm of the butanolic phase was measured. A calibration curve was performed using malondialdehyde (MDA), generated from 1,1,3,3-tetramethoxypropane (0.4–8 μM), as standard to express the absorbance changes as nmol MDA/μg protein.

### Western Blot

Western blot analyses were conducted as previously described [42]. Briefly, testes were quickly removed and stored at –70°C for western blot analyses. Tissue homogenates were prepared in a solution containing 50 mM Tris-HCl pH 7.5, 150 mM NaCl, 0.1% Triton X100, 0.5% sodium deoxycholate, 0.1% SDS, 1 mM PMSF, 5 μg/ml leupeptin, and 5 μg/ml aprotinin. After removal of cell debris by centrifugation, the protein concentration of the cell lysate was determined. The homogenates were combined with loading buffer containing 4% SDS, 20% glycerol, 10% β-mercaptoethanol, 125 mM Tris, (pH 6.8), and boiled at 100°C for 5 min. Protein samples (50 μg) were separated by 10% SDS-PAGE, and the separated proteins transferred to a PVDF membrane. Blots were incubated with primary antibodies (1:250 diluted rabbit anti-AR (C-19), Santa Cruz Biotechnology, Inc; 1:250 diluted rabbit anti-LHR (H-50), Santa Cruz Biotechnology, Inc; 1:250 diluted rabbit anti-FSHR (H-190), Santa Cruz Biotechnology, Inc; 1:500 diluted rabbit anti-TH, Pel Freez, USA; 1:1000 diluted rabbit anti-PCNA, Abcam, UK; 1:1000 diluted rabbit anti-BAX, Cell Signaling Technology, USA; 1:6000 diluted mouse anti-tubulin, Sigma). Immune complexes were detected with anti-rabbit secondary antibodies and chemiluminescence reagents (Amersham, NJ, USA), and bands were visualized in a C-DiGit® Chemiluminescent Western Blot Scanner (LI-COR Biosciences). The resulting images were quantified with ImageJ (NIH) software. Then, membranes were stripped and reprobed with monoclonal antibody against α-tubulin (1:3000, Sigma, USA) to confirm equal loading and transfer of samples.

### Real time PCR

Total testicular RNA was extracted with TRIzol (Invitrogen, USA) according to the manufacturer’s instructions. Total RNA (1 μg) was treated with DNAseI (Invitrogen, USA) and used for reverse transcription in a 20 μl final volume containing M-MLV reverse transcriptase (Promega, 200 U/μl, USA), and random hexamer primers (Biodynamics, USA). Reverse transcribed cDNA was employed for quantitative polymerase chain reaction (PCR) reactions using SYBR Green PCR Master Mix and specific primers (Table 1) in a Stratagene MPX500 cycler (Stratagene, USA). Data from the reaction were collected and analyzed by the complementary computer software (MxPro3005P v4.10 Build 389, Schema 85). Melting curves were run to confirm specificity of the signal. Relative quantitation of gene expression was calculated using standard curves and normalized to GAPDH in each sample.

**Table 1:** Primer sequences.

Gene	Acc. Number	Primer forward 5’-3’	Primer reverse 5’-3’
*Drd1a*	NM_010076	TTCTTCCTGGTATGGCTTGG	GCTTAGCCCTCACGTTCTTG
*Drd2*	NM_010077	TATGCCCTGGGTCGTCTATC	AGGACAGGACCCAGACAATG
*Adora1*	NM_001039510	TAGACAGTTCAGGTGGCCAG	AGTACATTTCCGGGCACAGA
*Adora3*	NM_009631	TCAGCCTGCAAGTCAAGATG	CAGCAAAGGCCCAAGAATAG
*Dazl*	NM_010021	AATGTTCAGTTCATGATGCTGCT	TGTATGCTTCGGTCCACAGACT
*Sod1*	NM_011434	AAAGCGGTGTGCGTGCTGA	CAGGTCTCCAACATGCCTCT
*Cat*	NM_009804	GCAGATACCTGTGAACTGTC	GTAGAATGTCCGCACCTGAG
*Gpx*	NM_008160	CCTCAAGTACGTCCGACCT	CAATGTCGTTGCGGCACAC
*Gapdh*	NM_008084	AGTGCCAGCCTCGTCCCGTAG	GTGCCGTTGAATTTGCCGTGAGTG

### Statistical analysis

InfoStat 2010 software (www.infostat.com.ar) was used for statistical analysis. Statistics were performed using one-way ANOVA followed by Bonferroni post hoc tests. Data were transformed when required. Data are expressed as the mean ± SEM. Differences were considered significant if p<0.05.

## Results

### 1. Chronic effects of caffeine, cocaine and their combination on testicular histology

**Fig 2:**
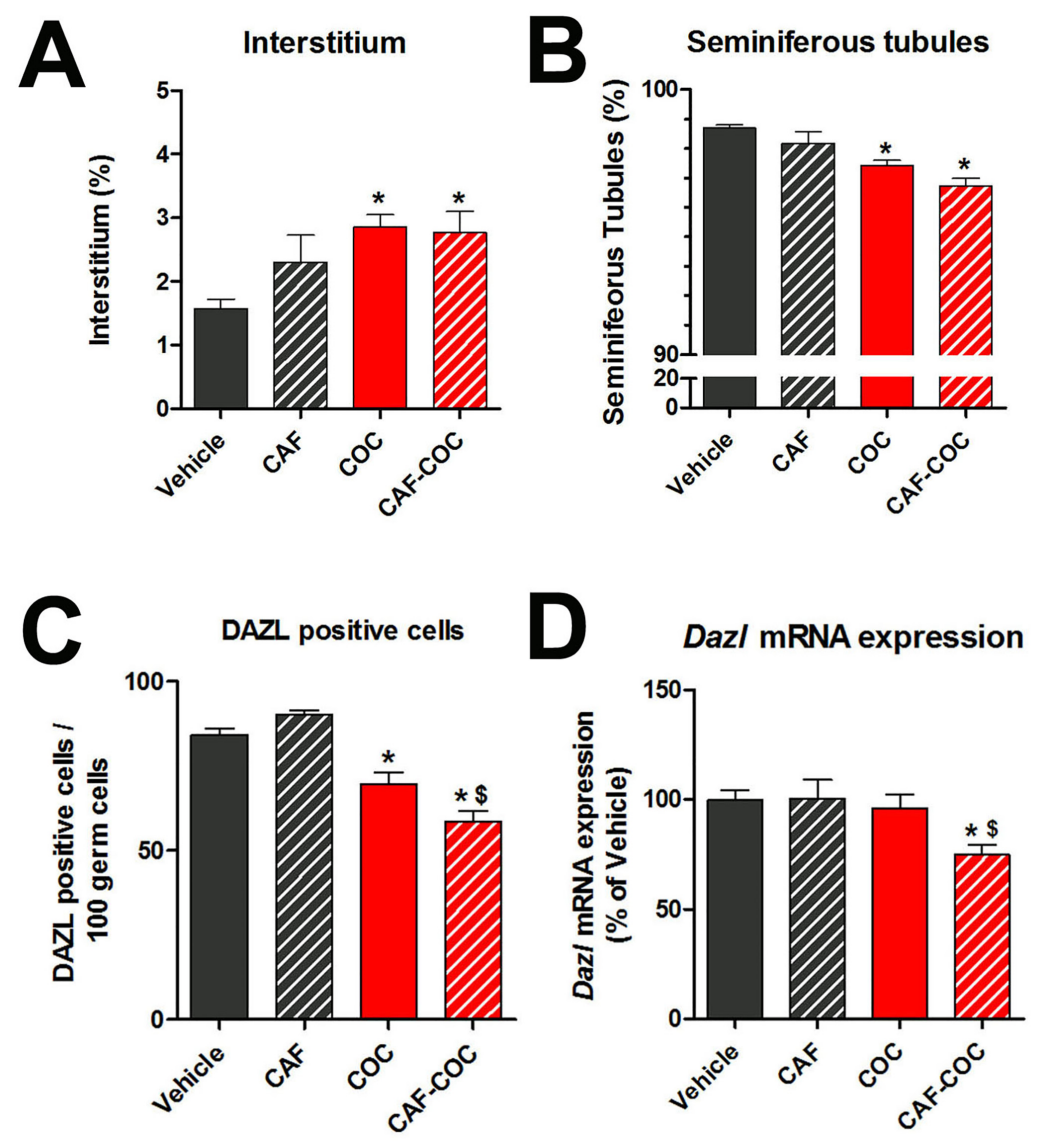
Effect of caffeine, cocaine, and their combination on testicular histology. Morphometric analysis of interstitial (A) and tubular (B) compartments. DAZL positive cell counts (C) and *Dazl* mRNA expression (D) in the testis from experimental groups. CAF: caffeine, COC: cocaine: ST: seminiferous tubule, I: interstitium. Values indicate mean ± SEM (N = 6). One way ANOVA- Bonferroni. * p<0.05 different from Vehicle; $ p<0.05 different from COC.

Testes are organs that, functionally and structurally, can be divided into two compartments: tubular, where the spermatogenic process takes place, and interstitial, were the androgen-producing Leydig cells are localized. Therefore, we first evaluated the effect of caffeine, cocaine and their combination on testicular compartments by morphometric analysis (Fig 2A and 2B). We found a significant decrease in the volume of seminiferous tubules and a concomitant increase in the interstitium in the COC and CAF-COC groups compared to Vehicle (p<0.05). To further analyze the seminiferous tubule volume reduction, we evaluated the spermatogonia marker DAZL by analyzing positive cell counts and *Dazl* mRNA expression [43] (Fig 2C and 2D). We found a significant decrease in the number of DAZL-positive spermatogonia in the COC and CAF-COC groups compared to Vehicle (p<0.01) (Fig 2C). Also, a significant decrease was observed in the CAF-COC compared to the COC group (p<0.05) (Fig 2D). *Dazl* mRNA expression was significantly decreased in the CAF-COC compared to Vehicle (p<0.05) (Fig 2D).

### 2. Chronic effects of caffeine, cocaine and their combination on LH and FSH receptors, proliferation and apoptotic markers

**Fig 3:**
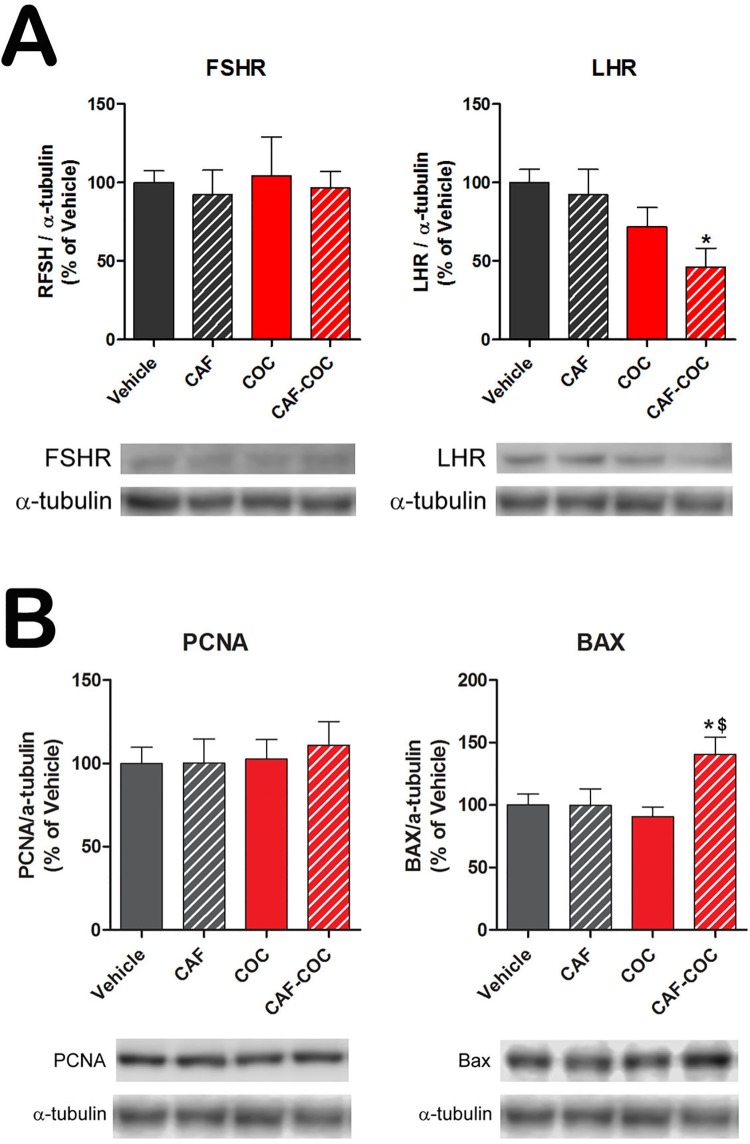
Effect of caffeine, cocaine, and their combination on protein expression of FSHR, LHR, and proliferation and apoptotic markers. **A)** Protein levels of hormone receptors FSHR and LHR and **B)** proliferation PCNA and pro-apoptotic BAX in the testes of experimental groups. CAF: caffeine; COC: cocaine. Values indicate mean ± SEM (N = 6). One way ANOVA- Bonferroni. * p<0.05 different from Vehicle; $ p<0.05 different from COC.

To further evaluate the effect of these treatments on testicular physiology, we analyzed the protein levels of the follicle stimulating hormone receptor (FSHR), indicative of the Sertoli cell number [44], and the luteinizing hormone receptor (LHR) indicative of Leydig cell function [45]. We found a significant decrease in LHR protein only in the CAF-COC group compared to Vehicle (p<0.05). No significant differences were detected in FSHR protein levels among groups. We also analyzed the testicular protein levels of the proliferating cell nuclear antigen (PCNA) and the pro-apoptotic marker BAX in all treatment groups (Fig 3B). We observed a significant increment in BAX protein in the CAF-COC group compared to Vehicle and COC groups (p<0.05). No significant differences were detected in PCNA protein levels among groups.

### 3. Chronic effects of caffeine, cocaine and their combination on testicular lipid peroxidation and antioxidant enzymes mRNA expression

**Fig 4:**
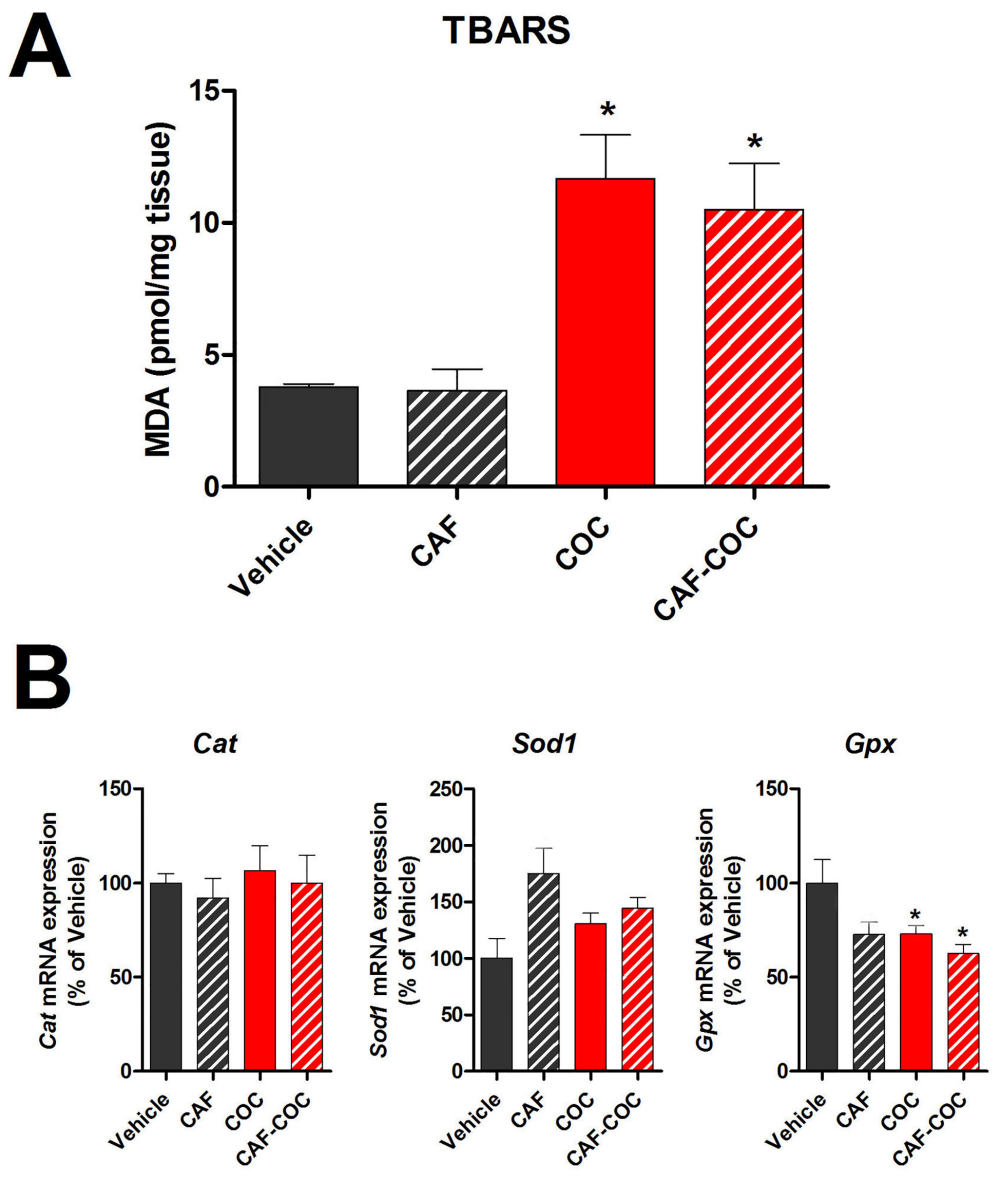
Effect of caffeine, cocaine and their combination on testicular lipid peroxidation and antioxidant enzymes mRNA expression. **A)** TBARS assay (N = 3–4). **B)** Antioxidant enzymes Catalase (*Cat*), superoxide dismutase (*Sod1*) and glutathione peroxidase (*Gpx*) mRNA expression (N = 5–6). CAF: caffeine; COC: cocaine. Values indicate mean ± SEM. One way ANOVA- Bonferroni. * p<0.05 different from Vehicle.

In order to determine testicular ROS levels after different treatments, lipid peroxidation by TBARS assay and mRNA expression of antioxidant enzymes were evaluated (Fig 4). COC and CAF-COC groups showed an increase in TBARS production (MDA) compared to Vehicle (p<0.05), indicative of an increase in ROS levels in cocaine-treated mice (Fig 4A). Fig 4B shows the mRNA expression of catalase (*Cat*), superoxide dismutase 1 (*Sod1*), and glutathione peroxidase (*Gpx*). *Gpx* mRNA showed a significant decrease in the COC and CAF-COC groups compared to Vehicle (p<0.05). No differences were detected in *Cat* and *Sod1* mRNA expression among groups.

### 4. Chronic effects of caffeine, cocaine and their combination on testicular dopaminergic and adenosinergic markers

**Fig 5:**
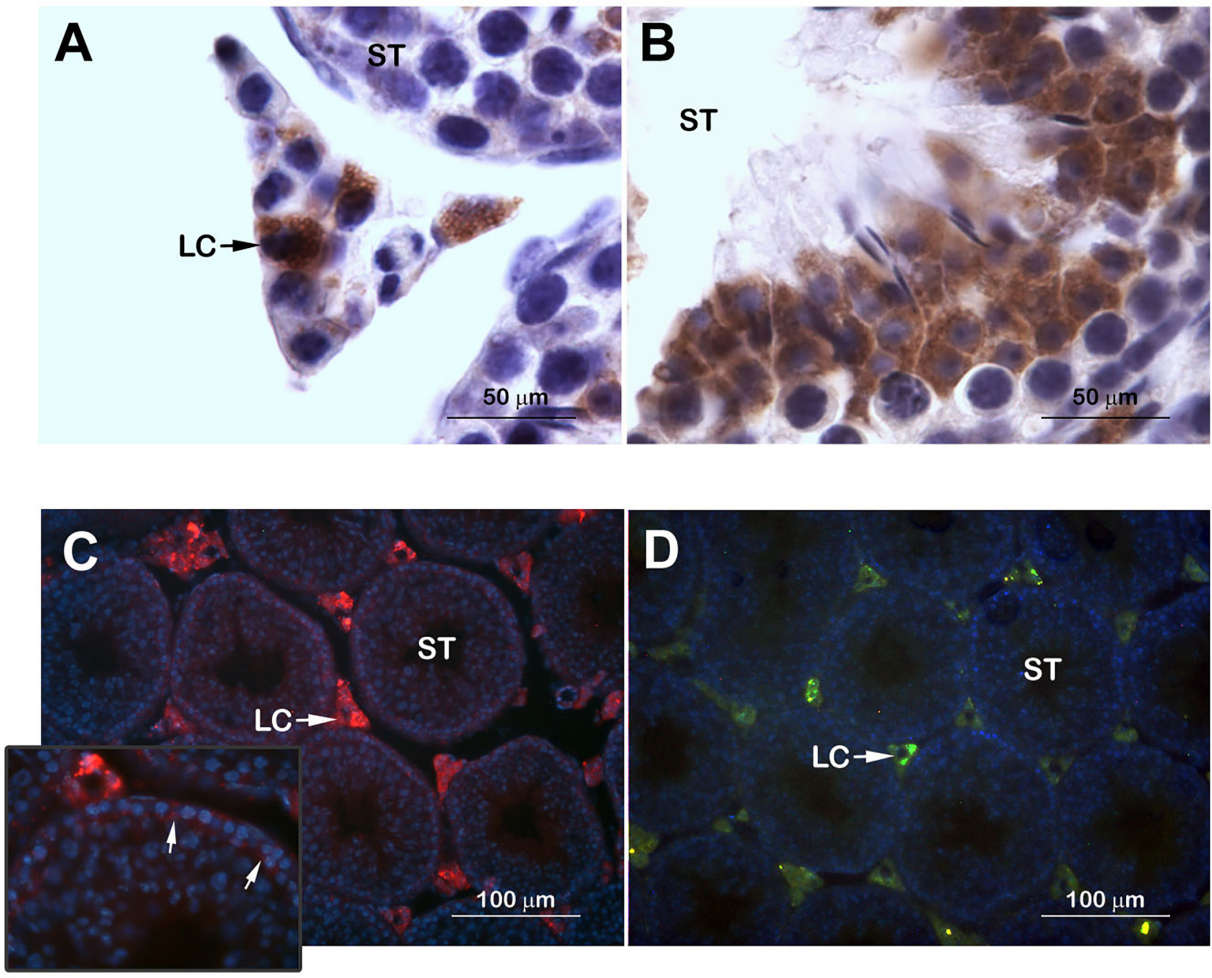
Testicular expression of TH and dopamine receptors. Testicular expression and cellular localization of the rate-limiting enzyme of catecholamine synthesis TH (**A, B**) as well as dopamine receptors DRD1 (**C)** and DRD2 (**D**) in testicular tissue of naïve mice. ST: seminiferous tubule; LC: Leydig cell; Inset arrows: DRD1 expression in spermatogonia nearest the basal lamina of the seminiferous tubules.

As a first approach to study possible local dopaminergic targets of psychostimulant actions, we evaluated testicular expression and cellular localization of the enzyme TH as well as DRD1 and DRD2 in naïve mice. TH protein immunolocalized in the cytoplasm of Leydig as well as in germ cells in the seminiferous tubules (Fig 5A and 5B). Interestingly, the expression of TH was mainly observed in meiotic germ cells. No TH expression was observed in the spermatogonia located near the basal lamina of the seminiferous tubule or Sertoli cells. To evaluate DRD1 and DRD2 testicular expression we used transgenic BAC-Drd1a-tdTomato and D2R-eGFP mice (Fig 5C and 5D). Both DRD1 and DRD2 promoter expression was detected in Leydig cells. DRD1 promoter expression was also observed in the spermatogonia near the basal lamina of the seminiferous tubules (Fig 5C inset).

**Fig 6:**
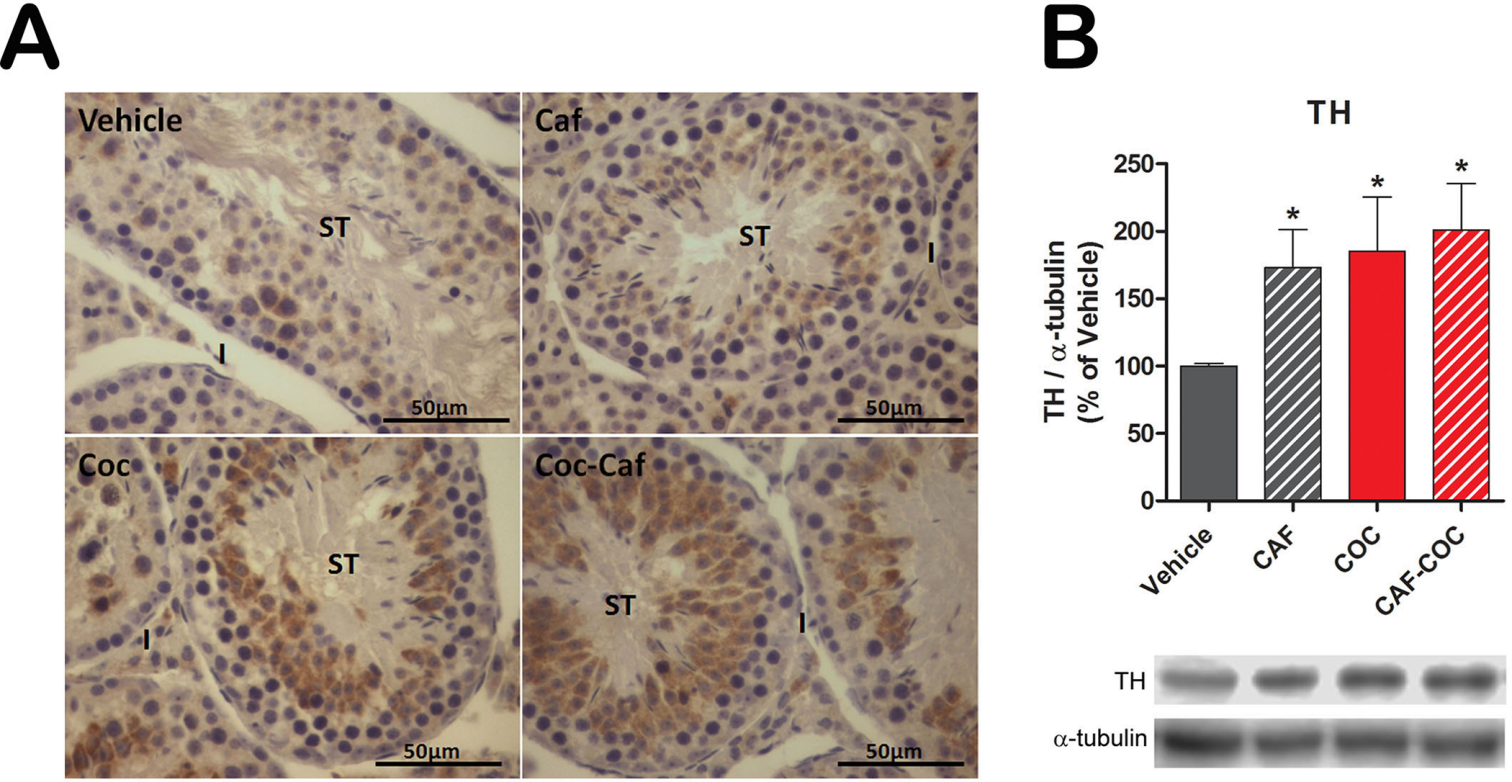
Testicular TH protein expression after caffeine, cocaine and their combination. **A)** TH immunostaining. **B)** TH protein levels evaluated by western blot. CAF: caffeine; COC: cocaine; I: interstitium; ST: seminiferous tubules; TH: tyrosine hydroxylase. Values indicate mean ± SEM (N = 5–6). One way ANOVA- Bonferroni. * p<0.05 different from Vehicle.

We also evaluated TH expression and protein levels in the experimental groups by immunohistochemistry and western blot, respectively. We found an increase in TH immunostaining (Fig 6A) and protein levels (Fig 6B) in the CAF, COC and CAF-COC groups compared to Vehicle (p<0.05).

**Fig 7:**
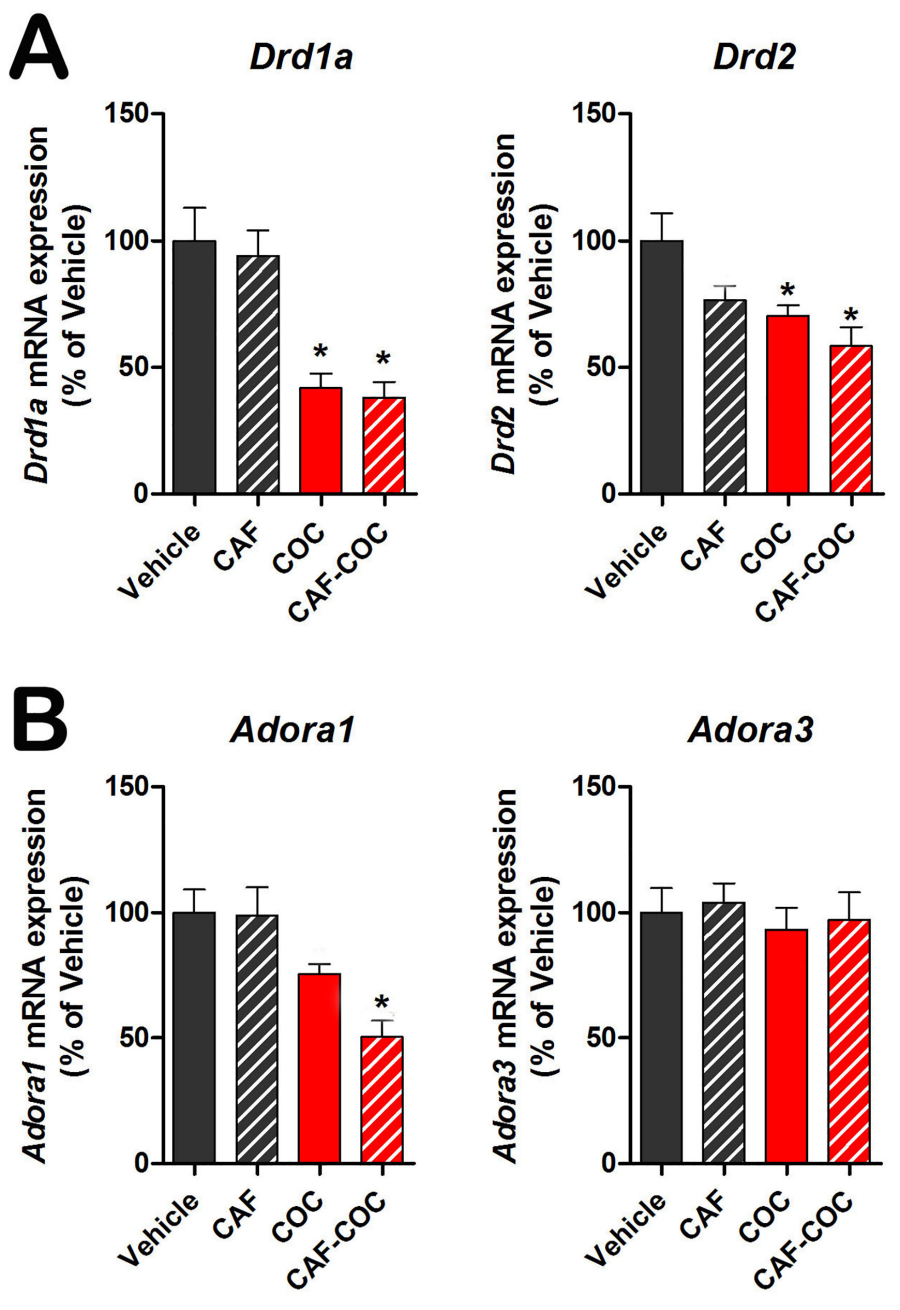
Testicular mRNA expression of dopamine and adenosine receptors after caffeine, cocaine and their combination. CAF: caffeine; COC: cocaine. Values indicate mean ± SEM (N = 6). One way ANOVA- Bonferroni. * p<0.05 different from Vehicle; $ p<0.05 different from COC.

Finally, we evaluated DA and adenosine receptor gene expression (*Drd* and *Adora*) after each treatment (Fig 7). *Drd1a* and *Drd2* mRNA expression was significantly decreased in the COC and CAF-COC groups compared to Vehicle (p<0.05) (Fig 7A). *Adora1* mRNA expression was significantly decreased in the CAF-COC group compared to Vehicle (p<0.05). No differences were detected in *Adora3* mRNA expression among groups (Fig 7B). We did not detect the presence of *Adora2a* or *Adora2b* mRNA transcripts.

## Discussion

The results presented here provide evidence of significant testicular alterations mediated by the administration of caffeine, cocaine and their combination, and highlight a potential role of the local dopaminergic system in testicular toxicity induced by psychostimulants. The interaction between caffeine and cocaine is relevant in terms of the frequency with which these two drugs are found in the products that reach consumers. Forensic analyses of seized illicit cocaine samples usually report variable quantities of caffeine [22]. Another issue relevant to this study is “coca paste”, an intermediate product of the cocaine extraction process from coca leaves commonly consumed in South America, which, in addition to a high content of cocaine base, is typically adulterated with caffeine [21]. In the present study we explored the effects of caffeine and cocaine treatments on testicular physiology, and also the potential additive/synergistic effects of the cocaine and caffeine combination by using psychostimulants doses that were previously shown to induce additive effects in the CNS [40]. We found that mice treated with cocaine manifested histological testicular alterations that were absent in mice treated with caffeine alone. Cocaine treatments reduced the volume of the seminiferous tubule with a concomitant reduction in DAZL-positive cells. We evaluated DAZL since it has been pointed out the importance of this protein function in spermatogonia meiotic division and differentiation [46,47]. We believe that our results (morphometry + DAZL-positive cell counts + *Dazl* mRNA) suggest a scenario where psychostimulants can induce germ cell loss. Importantly, the caffeine-cocaine combination induced lower DAZL-positive cell counts than cocaine-alone, indicating that caffeine was able to potentiate cocaine-induced germ cell loss. Accordingly Pires et al. [48] reported that crack cocaine induced a reduction in the number of germ cells and Sertoli cells, leading to a reduction of the tubular diameter in mouse testis. However, we did not detect changes in FSHR protein expression, which is considered a marker of Sertoli cell number [44], probably indicating that Sertoli cell number may not be altered under our experimental conditions.

Cocaine administration has been associated with hypothalamic-pituitary-gonadal axis dysregulation, with an increase in LH levels of unclear significance in men [49]. In addition, it has been reported that cocaine can reduce blood testosterone levels in rats and human consumers [50,51,3]. In our study, we observed that the expression of LHR protein was reduced in mice treated with the combination of caffeine and cocaine. Since LH is the main hormone regulating the testosterone production by Leydig cells, we speculate that chronic co-administration of both psychostimulants could negatively modulates the steroidogenic activity with a direct impact on the spermatogenic process.

ROS are free radicals that include superoxide anion, hydrogen peroxide, hydroxyl radical, lipid hydroperoxides, peroxyl radicals and peroxynitrite [16] having both beneficial and harmful roles depending of their concentration and duration of exposure [52]. In particular, lipid peroxidation is considered to be the main mechanism by which ROS can induce tissue damage leading to impaired cellular function in the testis [52,53]. It has been reported that prolonged exposure to cocaine leads to increased ROS production, germ cell apoptosis, and fibrosis of the seminiferous tubules in the male rat [54,6,51,48]. Accordingly, we observed that cocaine treatments induced TBARS production indicative of increased lipid peroxidation. Moreover, cocaine treatments reduced the mRNA levels of glutathione peroxidase, which is the antioxidant enzyme responsible for scavenging lipid peroxidation. Interestingly, caffeine on its own neither increased lipid peroxidation on its own, nor elicited potentiation of cocaine-induced ROS levels, suggesting that chronic caffeine treatment had no significant effects on testicular ROS levels. Additionally, there is evidence that caffeine can scavenge ROS and protect against lipid peroxidation [55,56]. It is important to point out that other reports using different cocaine protocols showed reduced glutathione peroxidase levels [6], and increased expression of pro-apoptotic markers such us DNA fragmentation, cytochrome c, caspase 3, and caspase 9 in rodent testis [57,51,48]. We did not observe changes in BAX expression in the cocaine-only treatment group, which could be related to the lower cocaine doses used in this study compared to previous ones [6,57,51]. In the present study, we detected an increase in the expression of BAX only after caffeine and cocaine combined treatment, further suggesting that caffeine may be able to potentiate cocaine-mediated pro-apoptotic effects.

In the nucleus accumbens and striatum, the reinforcing effect of cocaine is characterized by increased DA transmission that binds to DRs, which can induce DR downregulation [13,14]. Also, DAT blockade by cocaine leads to a reduction in DA intracellular concentrations and enhanced TH levels at the dopaminergic terminal [58]. As previously mentioned, increased DA transmission is an important component of the mechanisms that underlie psychostimulant neurotoxicity [16]. DA-induced cell death is thought to involve intraterminal DA autoxidation and generation of ROS, quinones, and semiquinones, with subsequent induction of neuronal apoptosis [16,42]. Regarding the dopaminergic system in the testis, data concerning the sites of biosynthesis and response of DA have been poorly investigated. TH has been detected in the interstitial compartment of the testis including Leydig cells and elongated neuron-like cells both in humans and monkeys [27,30,31]. In addition, only the DRD2 has been localized in rodent and human testicular germ cells [32]. It is important to point out the work done by James et al. [29] that showed, using viral retrograde tracing, the progression of the virus from testicular TH-expressing nerve fibers to dopaminergic brain areas such as the substantia nigra, indicating the existence of a direct brain-testicular pathway that controls testicular function.

In the present study, we showed that the toxic effects of cocaine on mouse testicular tissue occur in parallel with alterations in the local dopaminergic system. To our knowledge, this is the first report showing the presence of TH in the cytoplasm not only in Leydig cells, but also in meiotic germs cells within seminiferous tubules. Moreover, using transgenic BAC-Drd1a-tdTomato and D2R-eGFP mice, we report for the first time the presence of DRD1 and DRD2 in testicular mouse Leydig cells. Interestingly, the presence of DRD1 was also detected in the spermatogonia located nearest the basal lamina of the seminiferous tubules, which did not show TH staining. These cells are likely to be mitotically active spermatogonia. Our data show that the dopaminergic components are widely distributed in the testis indicating a possible role in the modulation of spermotogenic and steroidogenic processes by DA. We also observed that cocaine-only and combined treatment with caffeine induced downregulation of *Drd1* and *Drd2* mRNA expression in the testis. These results only indicate alterations at the transcriptional level and should then be interpreted with caution since protein levels may not follow the same trend. Still, our results on DRs gene expression alterations together with increases in TH levels, show a similar pattern to cocaine actions on DA pathways on the CNS [13,14,58].

To date, little is known about adenosine receptor function in testicular tissue. Studies in rat testis showed that the A3 receptor is expressed in spermatocytes and spermatids, and the A1 receptor is confined to Sertoli cells, suggesting that these receptors regulate male reproduction [33,59]. It has been shown that the A1 receptor contributes to the cytoprotective action of adenosine under oxidative stress conditions [60]. Nie et al. [61] have reported that ROS increase the expression of the A1 receptor, enhancing the cytoprotective role of adenosine. In the present study, we analyzed the effects of caffeine, cocaine, and their combination on A1 and A3 receptor gene expression. We show that cocaine and caffeine co-administration reduced A1 receptor mRNA expression. We hypothesize that combined treatment may have interfered with the protective role of this receptor in cocaine-induced oxidative stress.

Caffeine is the world’s most popular psychoactive drug, usually found in a range of commercially available products such as energy drinks that may be used in combination with other drugs such as alcohol and cocaine [62]. Recently, it was reported that high doses of caffeine altered endogenous testosterone secretion and reduced the sensitivity of Leydig cells to gonadotrophic stimulation [63]. Here, we report for the first time that caffeine by itself has specific testicular actions on dopaminergic markers, shown in the upregulation of TH protein expression. These results demonstrate that caffeine not only can potentiate the toxic effects of cocaine on testicular physiology, but it also has effects on its own linked to testicular DA system regulation.

## Concluding remarks

To our knowledge, this is the first study reporting actions of cocaine and caffeine on the local dopaminergic system in the testis, with a similar pattern to what it was previously reported for these two substances in the CNS. Furthermore, our study highlights a probable role of toxic events linked to testicular DA dysregulation that could influence epigenetic mechanisms contributing to paternal transmission of addiction traits. Further studies are warranted in order to expand the effect of components of the dopaminergic system on epigenetic regulators in testicular tissue.
